# Index

**DOI:** 10.1080/07853890.2021.1903745

**Published:** 2021-09-28

**Authors:** Ana Catarina Ramos, Catarina Godinho, A. P. Alves de Matos

The 4th edition of the CiiEM Congress “HEALTH, WELL-BEING AND AGEING IN THE XXI CENTURY” followed the trend of previous editions and kept the open and demanding spirit of Egas Moniz scientific dynamics, with the participation of a 15 high-quality communications from 15 world-renowned speakers, and about 200 communications focussing on subjects of importance to the scientific activity of Egas Moniz. This spreads across the biomedical and human sciences.

The Congress was held in the Egas Moniz University Campus at Monte de Caparica, near Lisbon, south of the Tagus river, from 3–5 June 2019.

The Congress was instrumental in bringing Egas Moniz research into contact with a broader audience, to learn new perspectives and to hear of cutting-edge scientific advances from international participants.

Massive participation of students demonstrated their interest in scientific activity, and paved the way for further initiatives to bring science, and its methods and didactic influence, to undergraduates, opening new perspectives to them for academic activities and lifelong learning.

The congress also made a valuable contribution in disseminating the scientific activity of Egas Moniz and its partner institutions, and in setting the stage for further developments in the forthcoming years.

## Funding

This work was financed by national funds through the FCT – Foundation for Science and Technology, I.P. (Portugal), under the project UIDB/04585/2020.

